# Delft Neighbors—Vermeer and van Leeuwenhoek

**DOI:** 10.3201/eid3206.AC3206

**Published:** 2026-06

**Authors:** Lesli Mitchell

**Affiliations:** Centers for Disease Control and Prevention, Atlanta, Georgia, USA

**Keywords:** bacteria, microscope, the Netherlands, Johannes Vermeer, Antonie van Leeuwenhoek, art-science connection

**Figure:**
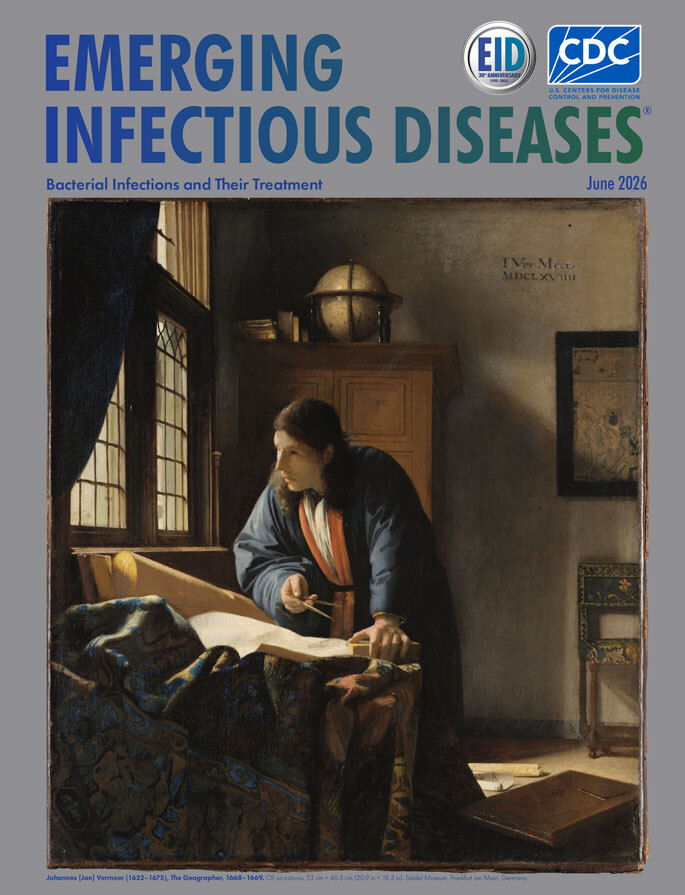
**Johannes [Jan] Vermeer (1632–1675),** *The Geographer,* 1668–1669. Oil on canvas. 53 cm × 46.5 cm (20.9 in × 18.3 in). Städel Museum, Frankfurt am Main, Germany. https://www.johannesvermeer.org.

Jan Vermeer’s *The Geographer*, featured on the cover of this issue, is widely considered to be a pendant of *The Astronomer*, thematically similar and intended to be displayed together. EID featured *The Astronomer* on the cover of its January 2004 issue, with an accompanying essay by the journal’s esteemed Founding Managing Editor, Polyxeni Potter. An extensive background in art history coupled with her insightful writing have contributed to EID’s current reputation for its signature artwork.

In her essay, Potter noted that Vermeer is often linked to Antonie van Leeuwenhoek (sometimes spelled Antoni or Anthony), whose portrait was featured in EID’s January 2024 cover essay by Byron Breedlove. Van Leeuwenhoek was a Dutch inventor who discovered through his microscope the cellular nature of spermatozoa and bacteria and was skilled in navigation, astronomy, and mathematics. There is also speculation that he was the model for the subject of both paintings.

This irresistible notion that Vermeer and van Leeuwenhoek were connected in some way continues today and, considering their backgrounds, it is hard to believe they would *not* have known each other. Born only a few days apart in Delft in October 1632 and baptized in the same church, they lived within a few minutes’ walk of each other. Delft was not so large a town that its most famous artists and scientists would be unknown to each other. The two men had friends in common, including Constantijn Huygens, an influential diplomat, artist, and poet who possessed a keen interest in microscopy and the natural sciences. From the windows of City Hall, where van Leeuwenhoek worked as a city official, one could see the front of Mechelen Inn, the tavern Vermeer owned.

Fortunate to live and work during a period referred to as the Dutch Golden Age, Vermeer and van Leeuwenhoek embraced the mindset of that time—one of scientific exploration and discovery. Their interests in optics and light are reflected in their work. Vermeer’s *The Geographer* is a carefully composed allegorical image of scientific progress. It includes a host of objects that emphasize not only the focus on scientific exploration but also specific Dutch contributions. The map on the wall is so precisely detailed that it can be identified: it is one of the Pascaarten, printed by Willem Janszoon Blaeu in Amsterdam in the early 17th Century. The globe is also an acknowledgment of Dutch scientific achievement: it’s a model of one made in 1600 by Flemish engraver and cartographer Jodocus Hondius the Elder in Amsterdam. He sold the globe together with its celestial counterpart, featured in Vermeer’s *The Astronomer*. The pair of globes forms a cosmological whole, unifying heaven and earth. The geographer’s globe is also turned to show the Indian Ocean, the route used by ships of the Dutch East India Company. The youth and energy of the scholar in both paintings convey the excitement of the times. The geographer seems to be on the brink of an insight, holding a divider while referencing a scholarly text, his face illuminated by the light through the window.

Like Vermeer, van Leeuwenhoek was fascinated by light, and his interest in optics led to his experiments in lenses to improve clarity and magnification. van Leeuwenhoek learned glass-blowing techniques and used them to make numerous lenses that could magnify 500 times, enabling him to visualize bacteria. He did not share the techniques he used, and what was a mystery then is still a mystery today, but his lenses were far superior to others made during that time. Versions of a microscope appeared as early as 1595, but van Leeuwenhoek refined it in devising a high-powered microscope that magnified 200–500 times, far greater than any previous microscope. Through his high-powered microscope, he made a host of discoveries: “animalcules” (tiny animals) swimming in a drop of pond water, blood cells, bacteria, free-living and parasitic microscopic protists, sperm cells, microscopic nematodes and rotifers, and more. His discoveries opened a new world of microscopic life to scientists.

Recognizing how these visionary men of Delft contributed to the Dutch Golden Age, it’s tempting to connect them, and many scholars have done so, suggesting that they knew each other and even influenced each other’s work. The speculation that van Leeuwenhoek was the model for the young man in both *The Astronomer* and *The Geographer* is difficult to prove, however; the scholar in Vermeer’s paintings bears little resemblance to the portrait of van Leeuwenhoek painted by Jan Verkolje in 1686 (see page 1019). van Leeuwenhoek is also one of the figures in the 1681 painting *Anatomic lesson of Cornelis van’s-Gravensande* by Cornelis de Man (Figure). He sat for the group portrait for the surgeon’s guild at the age of 49 and is featured near the top left of the painting. The resemblance is clear in those two paintings, but it is arguable whether they resemble the young man featured in Vermeer’s works.

**Figure:**
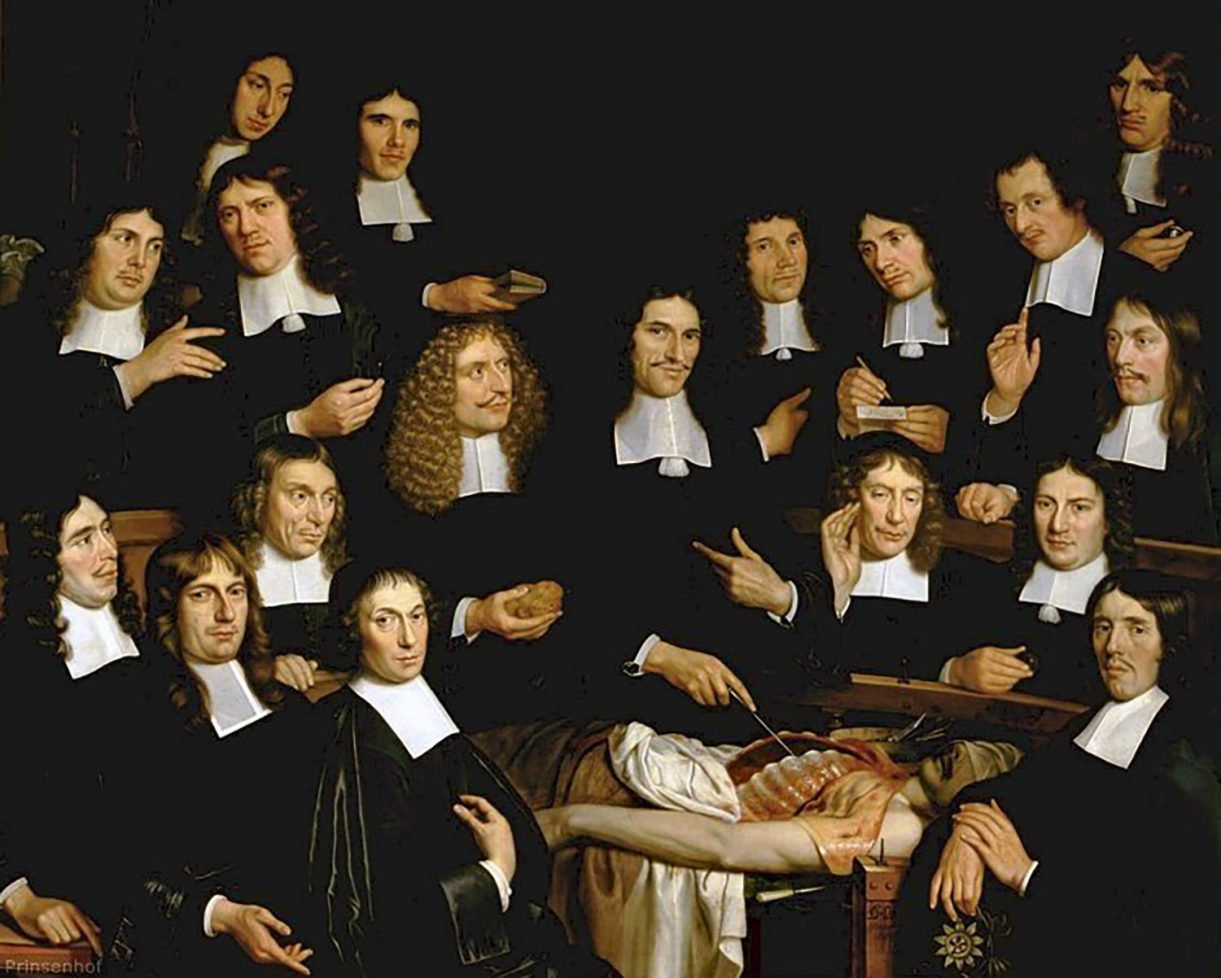
Cornelis de Man, *Anatomic lesson of Cornelis van’s-Gravensande*, 1681. Oil on canvas. 173.0 × 212.0 cm (68.1 in × 83.5 in). Collection of the Museum Prinsenhof Delft, Delft, the Netherlands. Gift of the Reinier de Graaf Groep; photograph by Tom Haartsen.

In fact, the only solid evidence linking both men is a legal one: van Leeuwenhoek was appointed as executor of Vermeer’s estate after the artist’s early death at the age of 43. That fact suggests a connection, but the appointment does not mean that the men knew each other personally; curator duties were part of van Leeuwenhoek’s job with the magistrate’s court, and the Vermeer case was the fifth of about 10 known cases. If the two men did have a relationship or friendship, it is hard to see in van Leeuwenhoek’s treatment of Vermeer’s widow, Catharina Bolnes, in the dispensation of the estate. As art historian Gary Schwartz notes, “The curator of an estate has permissible options that can benefit the heirs to a bankrupt estate. Van Leeuwenhoek did not employ them.” Despite Vermeer being bankrupt at the end of his life, his widow hoped to keep *The Art of Painting* within the family as a memento. van Leeuwenhoek noticed the omission of the painting in the home’s inventory and made sure it was auctioned off to pay Vermeer’s creditors.

As James Galbraith notes in his blog at the Corning Museum of Glass, “Until someone in Delft discovers a letter in their attic from Vermeer to van Leeuwenhoek or a similarly revealing document comes to light, we will never know with certainty whether or not the two men knew each other.” Even so, a relationship between the two men need not be romanticized to appreciate their influence in the city of Delft and the lasting contributions they made to art and science. The tantalizing question of their connection, even to this day, is a testament to their legacy and to the enduring esteem both men have in the public imagination. This issue’s theme on bacterial infections and their treatment includes more information on van Leeuwenhoek, including a book review of *Myriad, Microscopic and Marvelous: The World of Antoni van Leeuwenhoek*. The articles in this issue highlight that we not only see microorganisms more clearly now but also understand their role in disease and how to treat them when they are human pathogens.
